# Comparable Specimen Collection from Both Ends of At-Home Midturbinate Swabs

**DOI:** 10.1128/JCM.03073-20

**Published:** 2021-04-20

**Authors:** Melissa Truong, Brian Pfau, Evan McDermot, Peter D. Han, Elisabeth Brandstetter, Matthew Richardson, Ashley E. Kim, Mark J. Rieder, Helen Y. Chu, Janet A. Englund, Deborah A. Nickerson, Jay Shendure, Christina M. Lockwood, Eric Q. Konnick, Lea M. Starita

**Affiliations:** a Brotman Baty Institute for Precision Medicine, Seattle, Washington, USA; b University of Washington, Seattle, Washington, USA; c Seattle Children’s Research Institute, Seattle, Washington, USA; d Howard Hughes Medical Institute, Seattle, Washington, USA; University of Iowa College of Medicine

**Keywords:** COVID-19, at-home, self-swab

## LETTER

**FIG 1:**
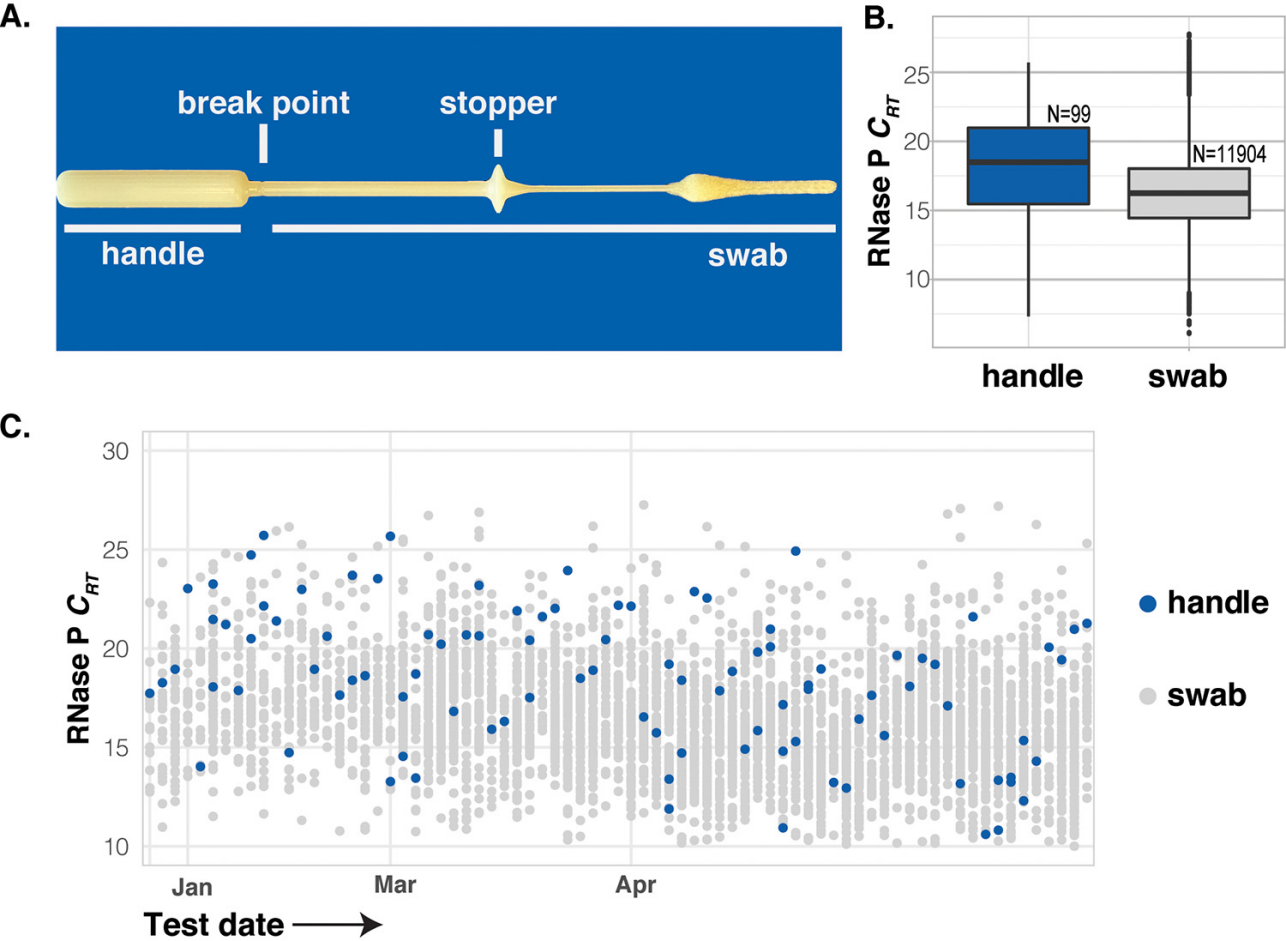
(A) A midturbinate swab (Copan 56380CS01), underlined handle or swab, was placed in UTM by participants. (B) *C_rt_* values from all samples with RNase P detected; dashed line indicates detection limit. (C) *C_rt_* values for human RNase P among batches of specimens (arranged on the *x* axis by date) where at least one handle specimen was used.

At-home respiratory specimen collection for pathogen testing enables community sampling. Furthermore, it requires neither a health care worker’s time nor personal protective equipment, and symptomatic individuals can continue to self-isolate. However, questions remain as to whether unsupervised upper respiratory specimen collection by individuals in their homes reliably produce specimens that are of high enough quality for pathogen testing. From October 2019 through May 2020, the Seattle Flu Study (1, 2) and the greater Seattle Coronavirus Assessment Network (SCAN; scanpublichealth.org) screened 16,785 midturbinate swabs that were self-collected by participants at home for respiratory pathogens. The at-home kits contained a flocked, midturbinate swab (Copan 56380CS01 or 56750CS01), either adult or pediatric, a tube of universal transport media (UTM), and instructions on how to self-collect a specimen or collect a specimen for a child and return it to the lab (2). Of the kits distributed to individuals in the Seattle metropolitan area, most resulted in swabs returned appropriately according to the instructions in the kit, but 138/16,785 (0.8%) kits were returned to the lab with the swab handle in the UTM tube rather than the swab itself. The swab handle is nontapered, hard plastic with decreased surface area compared with the flocked end of the swab (Fig. 1A). We were puzzled by this phenomenon and sought to evaluate whether handle-collected specimens were comparable to flocked swabs themselves for molecular pathogen detection. We also assessed demographic covariates associated with errors in swab collection.

Of the 16,782 specimens, 12,006 were analyzed for the presence of 24 respiratory pathogens using our TaqMan-based detection panel, including 99 of the 138 specimens collected with the handle (Table 1). Samples collected after 1 January 2020 were additionally tested for the presence of severe acute respiratory syndrome coronavirus 2 (SARS-CoV-2) using a separate reverse transcriptase PCR (RT-PCR) assay. As a quality-control metric to determine if a sufficient nasal specimen was collected for each sample, both assay platforms measured the amount of human RNase P. Specimens with RNase P relative cycle threshold (*C_rt_*) of >28 were considered to be a failed collection. The failure rate for all properly collected specimens was 2.0% (238/12,142). We expected a high failure rate for the handle-collected specimens, but only 2.9% (3/102) failed this quality-control metric, a nonsignificant difference (*P* = 0.46, Fisher’s exact test). The *C_rt_* values for human marker RNase P for handle-collected specimens were higher than those for properly collected specimens (Fig. 1B), with a mean *C_rt_* value of 16.32 (95% confidence interval [CI], 16.27 to 16.37) for swabs and 18.19 (95% CI, 17.43 to 18.96) for handles (*P* < 0.01). However, the *C_rt_* from handle-collected specimens generally fell within the same range and well below the failure threshold (Fig. 1C), showing that the handles were indeed collecting human cells. In addition, we identified multiple respiratory pathogens, including SARS-CoV-2, at similar rates of detection with both swabs and swab handles (*P* = 0.52) (Table 1).

**TABLE 1:** Detection rates of respiratory pathogens

Pathogen	No. (%) of respiratory pathogens	
	Handle present	Swab present
Adenovirus	1 (1.0)	77 (0.6)
Bocavirus	0 (0.0)	12 (0.1)
Enterovirus	1 (1.0)	29 (0.2)
Influenza A	4 (4.0)	394 (3.3)
Influenza B	0 (0.0)	250 (2.1)
Influenza C	0 (0.0)	0 (0.0)
Metapneumovirus	0 (0.0)	77 (0.6)
Parainfluenza	1 (1.0)	50 (0.4)
Parechovirus	0 (0.0)	0 (0.0)
Respiratory syncytial virus	2 (2.0)	116 (1.0)
Rhinovirus	6 (6.0)	620 (5.2)
SARS-CoV-2	1 (1.1)	119 (1.1)
Seasonal coronavirus	3 (3.0)	350 (2.9)
Chlamydia pneumoniae	0 (0.0)	8 (0.1)
Mycoplasma pneumoniae	0 (0.0)	28 (0.2)
Streptococcus pneumoniae	3 (3.0)	275 (2.3)

**FIG 2:**
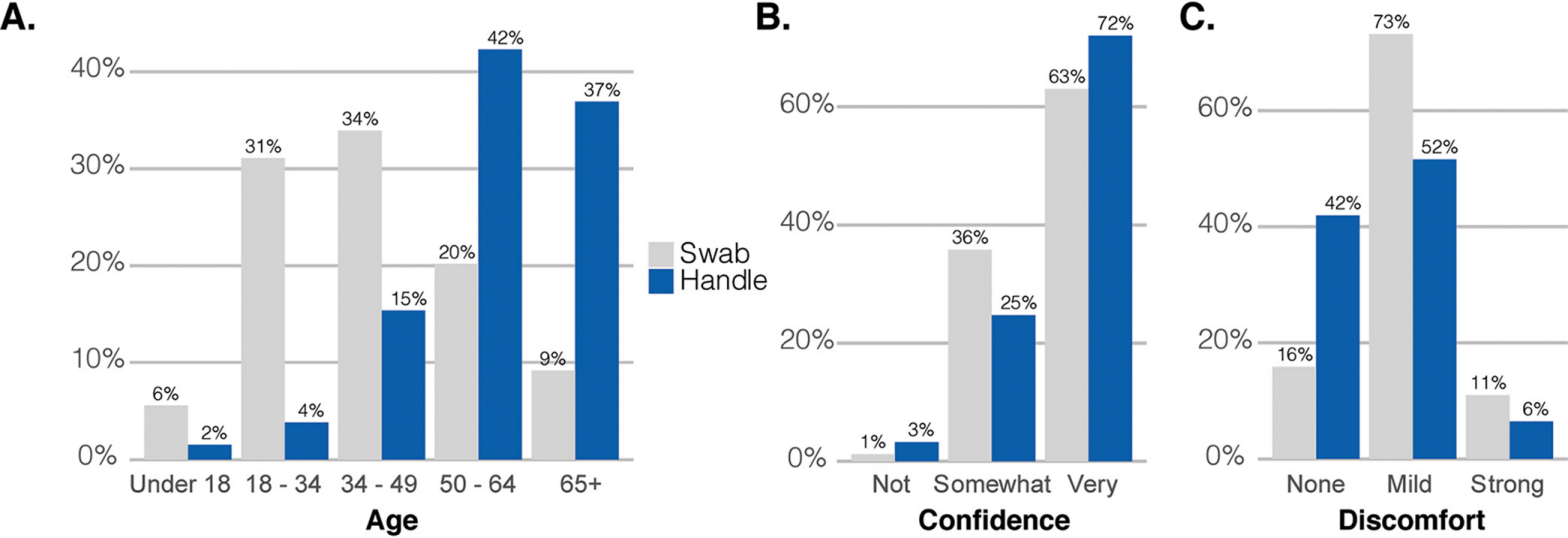
(A) Age of participants. (B) Self-reported confidence in specimen collection. (C) Self-reported discomfort during specimen collection by which end of the swab was used.

We examined the clinical data associated with the samples to determine which participants were more likely to collect a specimen with the handle. Participants who swabbed with the handle were more likely to be older (Fig. 2A), with a median age of 62 compared with 39 for those who followed the instructions (*P* < 0.01). There was no significant difference in handle use between men and women (*P* = 0.22) or across income brackets (supplemental material). Interestingly, participants who had erroneously used the handle were more confident that they had collected a quality specimen (Fig. 2B) (73% highly confident with the handle versus 62% with the swab, *P* = 0.02) and reported lower overall discomfort (Fig. 2C) (42% reported no discomfort with the handle versus 16% with the swab, *P* < 0.01). The greater reported comfort, combined with the larger size of the handles, suggests that these specimens were collected from the anterior nares rather than the midturbinate.

We investigated unanticipated operator error in two large studies employing at-home midturbinate swab collection and determined that participants who used the plastic handle rather than flocked swab to collect their sample and submit it to a laboratory were able to collect an adequate nasal specimen for molecular detection of respiratory pathogens. Like other studies (3), these results suggest that the use of specialty swabs may result in only marginal increases in pathogen detection. They also suggest that even if participants do not closely adhere to instructions, they can still collect a sample that is sufficient for the molecular detection of respiratory pathogens, including influenza and SARS-CoV-2.

The Seattle Flu Study received approval by the University of Washington’s Institutional Review Board (UW IRB; STUDY00006181), and informed consent was obtained prior to study enrollment. Participants joined SCAN as part of public health surveillance.

## Supplementary Material

Supplemental file 1
