# Supplementary material for: Comparable Specimen Collection from Both Ends of At-Home Midturbinate Swabs
Source: J Clin Microbiol. 2021 Apr 20;59(5):e03073-20. doi: 10.1128/JCM.03073-20 (PMC8091862; doi:10.1128/JCM.03073-20)
Supplement: Supplemental file 1 [file JCM.03073-20-s0001.pdf]

Supplementary Methods

Study description: The Seattle Flu Study Swab & Send program was IRB approved (STUDY00006181) and consent is obtained from participants or their guardians online at time of enrollment.The greater Seattle Coronavirus Assessment Network (SCAN) is a public health initiative under the direction of Public Health Seattle King County and permission is obtained from participants for molecular testing. For both studies, participants were sent a self-test kit to their home following online enrollment and answering a brief questionnaire. Participants collected their own mid-turbinate specimen, unsupervised, with written and video instructions (scanpublicheatlh.org). Instructions and materials were included for the participants to package and ship the sample according to IATA bio-specimen regulations. In January through March 22 2020, samples were shipped by US Postal Service and after March 23, samples were rapidly returned by courier service. An in-depth description of the Seattle Flu Study Swab & Send program can be found (1). SCAN is based on the Swab & Send program with improvements aimed at achieving greater geographic and demographic diversity.

Molecular testing: Specimens were shipped to the Brotman Baty Institute for Precision Medicine via commercial couriers or the US Postal Service at ambient temperatures and opened in a class II biological safety cabinet in a biosafety level-2 laboratory. When opening specimen packages and transferring samples, technicians recorded basic information, including participant compliance with labelling and packaging instructions. Sample entries with notes indicating that mistakes had been made by participants were manually curated to determine if they belonged in the handle or the standard swab cohort. Samples with both the swab and the handle in the UTM vial were excluded from both groups.

Two or three 650 µL aliquots of UTM were collected from each specimen and stored at 4˚C until the time of nucleic acid extraction, performed with the MagnaPure 96 small volume total nucleic acids kit (Roche).

Molecular assays were performed at the Northwest Genomics Center (Department of Genome Sciences, the University of Washington). Extracted nucleic acids were tested for the presence of 24 respiratory pathogens by TaqMan RT-PCR on the OpenArray platform (**Appendix Table 1**) and separate RT-PCR assay for SARS-CoV-2. For the Open Array pathogen panel, the extracted nucleic acid samples were added to a PreAmp reaction master mix containing TaqPath 1-Step RT-qPCR Master Mix CG, a custom TaqMan PreAmp oligonucleotide pool, and a spike-in control (TaqMan Universal Xeno RT control, ThermoFisher). The pre-amplification reactions were reverse transcribed and amplified for 14 PCR cycles according to the manufacturer's recommendations. The preamplified samples were diluted and added to Open Array Mastermix (ThermoFisher); the mix was then re-arrayed onto custom Open Array plates containing each RT-PCR assay in duplicate. RT-PCR was performed on a QuantStudio 12 (Applied Biosystems), with cycling parameters set according to the manufacturer's recommendations, for a total of 35 cycles. Positive and negative template controls were included in each extraction and PCR batch.

Table 1

Organism	Open Array Probe sets
Adenovirus	AdV_1of2, AdV_2of2
Seasonal Coronavirus	CoV_HKU1_CoV_NL63, CoV_229E_CoV_OC43
Influenza	Flu_A_H1, Flu_A_H3, Flu_A_pan, Flu_B_pan, Flu_C_pan
Respiratory Syncytial Virus	RSVA, RSVB
Parainfluenza virus	hPIV1_hPIV2, hPIV3_hPIV4

Metapneumovirus	hMPV
Enterovirus	EV_pan, EV_D68
Parechovirus	HPeV
Bocavirus	HBoV
Rhinovirus	RV_1of2, RV_2of2
Pneumoniae	S.pneumoniae, C.pneumoniae, M.pneumoniae

Data files from the Open Array were imported into and processed through our in-house LIMS system. Detection of each technical replicate is calculated independently using threshold values provided by ThermoFisher; the relative cycle threshold (C_{RT}) must be less than or equal to the C_{RT} threshold for each respiratory pathogen to be deemed present. Additionally, both the C_Q Conf and Amp Score (measures of the shape of the curve generated by Quantstudio 12 software) must be greater than or equal to separate thresholds for each respiratory pathogen to be flagged as detected. Samples with an RNAse P $C_{RT} > 28$ are considered failed. The failure rate for all properly-collected specimens versus handle-collected specimens was analyzed via R Studio (Version 1.2.5033) using Fisher's exact test.

SARS-CoV-2 was detected using a laboratory-developed test (LDT) or research assay. For the LDT, SARS-CoV-2 detection was performed using real-time RT-PCR with a probe sets targeting Orf1b and S with FAM fluor (Life Technologies 4332079 assays # APGZJKF and APXGVC4APX) multiplexed with an RNaseP probe set with VIC or HEX fluor (Life Technologies A30064 or IDT custom) each in duplicate on a QuantStudio 6 instrument (Applied Biosystems). The Northwest Genomics Center LDT is clinically certified by the state of Washington. The research assay employs only the Orf1b and RNaseP multiplexed RT-PCR in duplicate. Three or four replicates for RNase P and SARS-CoV-2 were required to have a cycle threshold (C_T) < 40

for a sample to be considered positive in the LDT or both replicates must be positive in the research assay. Specimens with two replicates with SARS-CoV-2 detected are considered inconclusive.

Data Analysis: Code and data to reproduce all figures and results can be found at https://github.com/bbi-lab/handle-vs-swab. RNAse P C_T values and participant demographics were analyzed in RStudio Version 1.2.5042. Replicate C_T values for RNAse P were averaged, and the mean C_T values for handles and swabs were compared using a two-tailed Welch's two-sample t-test. Pathogen detection rates were compared using a paired t-test.

Participant data were collected and managed using REDCap electronic data capture tools hosted at the University of Washington (2,3). REDCap (Research Electronic Data Capture) is a secure, web-based software platform designed to support data capture for research studies, providing 1) an intuitive interface for validated data capture; 2) audit trails for tracking data manipulation and export procedures; 3) automated export procedures for seamless data downloads to common statistical packages; and 4) procedures for data integration and interoperability with external sources. Participants measured their level of discomfort and confidence in their swab technique on a voluntary online survey taken at time of sample collection. The three levels of comfort and confidence, respectively, were each compared using a Fisher's exact test. Participant age was compared using a two-tailed Welch's two-sample t-test, and sex was compared with a Pearson's Chi-squared test with Yates' continuity correction.

References:

1. Kim AE, Brandstetter E, Graham C, Heimonen J, Osterbind A, McCulloch DJ, Han PD, Starita LM, Nickerson DA, Van de Loo MM, Mooney J, Rieder MJ, Ilcisin M, Fay KA, Lee J, Sibley TR, Bedford T, Englund JA, Boeckh M, Chu HY. 2020. Seattle Flu Study - Swab and Send: Study Protocol for At-Home Surveillance Methods to Estimate the Burden of Respiratory Pathogens on a City-Wide Scale. medrxiv;2020.03.04.20031211v1. Infectious Diseases (except HIV/AIDS). medRxiv.

2. Harris PA, Taylor R, Minor BL, Elliott V, Fernandez M, O’Neal L, McLeod L, Delacqua G, Delacqua F, Kirby J, Duda SN, REDCap Consortium. 2019. The REDCap consortium: Building an international community of software platform partners. J Biomed Inform 95:103208.

3. Harris PA, Taylor R, Thielke R, Payne J, Gonzalez N, Conde JG, Others. 2009. A metadata-driven methodology and workflow process for providing translational research informatics support. J Biomed Inform 42:377–381.
